# Interaction of *Candida* Species with the Skin

**DOI:** 10.3390/microorganisms5020032

**Published:** 2017-06-07

**Authors:** Andreas Kühbacher, Anke Burger-Kentischer, Steffen Rupp

**Affiliations:** 1 Department of Molecular Biotechnology, Fraunhofer Institute for Interfacial Engineering and Biotechnology, 70569 Stuttgart, Germany; andreas.kuehbacher@igb.fraunhofer.de (A.K.); anke.burger-kentischer@igb.fraunhofer.de (A.B.-K.); 2 Institute of Interfacial Process Engineering and Plasma Technology, University of Stuttgart, 70569 Stuttgart, Germany

**Keywords:** *Candida*, skin infection, 3D-tissue models, innate immunity, fibroblasts

## Abstract

The human skin is commonly colonized by diverse fungal species. Some *Candida* species, especially *C. albicans*, do not only reside on the skin surface as commensals, but also cause infections by growing into the colonized tissue. However, defense mechanisms at the skin barrier level are very efficient, involving residential non-immune and immune cells as well as immune cells specifically recruited to the site of infection. Therefore, the skin is an effective barrier against fungal infection. While most studies about commensal and pathogenic interaction of *Candida* species with host epithelia focus on the interaction with mucosal surfaces such as the vaginal and gastrointestinal epithelia, less is known about the mechanisms underlying *Candida* interaction with the skin. In this review, we focus on the ecology and molecular pathogenesis of *Candida* species on the skin and give an overview of defense mechanisms against *C. albicans* in this context. We also discuss new research avenues in dermal infection, including the involvement of neurons, fibroblasts, and commensal bacteria in both mouse and human model systems.

## 1. Dermal *Candida* Ecology and Epidemiology

Colonization of human skin with bacterial and fungal communities has been observed for decades, but much remains to be understood about host-fungus interactions at this surface. Nowadays, different fungi including *Malassezia*, *Cryptococcus*, *Rhodotorula*, and *Candida* species have been identified as human skin commensals. In 2013, Findlay and coworkers analyzed the fungal communities of various human skin sites of healthy individuals. The authors found that 11 out of 14 tested sites were predominantly colonized by *Malassezia* species, with diversity only at the species level, while fungal communities of three sites on the feet—namely planar heel, toenail, and toe web— were much more diverse [1]. It has also been shown that under conditions of primary immunodeficiencies, fungal diversity—and in particular the abundance of *Candida* species—on the skin increases [2].

Although fungi are part of the commensal skin microbiota, various species are also pathogenic. It has been estimated that 20–25% of the world population is affected by fungal skin infections [3]. Fungal skin pathogens can be divided into two classes: dermatophytes and yeast, with *Candida* species belonging to the latter. Among the 200 known *Candida* species, only a few, including *C. tropicalis*, *C. parapsilosis*, and *C. orthopsilosis*, commonly found on healthy skin, can become pathogenic [1,4]. *C. albicans* is the *Candida* species most often responsible for symptomatic skin infections [3]. Common symptoms of *Candida* skin infections include thickening of the skin, hyperkeratosis, and erythema [5]. 

Several physical and immunological factors influence *Candida* skin infections, which display a preference for occluded regions of skin where humidity and CO_2_ accumulate and the skin is constantly experiencing friction [6]. For example, such conditions can be found while infants are in diapers, where the combination of elevated pH and the presence of lipases and proteases from feces commonly leads to secondary *Candida* infections, mainly caused by *C. albicans* from the gastrointestinal tract, resulting in diaper dermatitis, as reviewed in [7]. Recurrent *Candida* infections of the skin and mucosal surfaces are referred to as chronic mucocutaneous candidiasis (CMC), which occurs mainly in individuals with primary or acquired immunodeficiencies [5], upon antibiotic treatment or injury with varying individual risk factors dependent on the epithelial surfaces [8]. Increased *Candida* colonization has also been associated with other skin disorders, including atopic dermatitis and psoriasis [9,10]. In addition to colonization, the risk for superficial *Candida* infections is increased for individuals suffering from psoriasis. In part, this may also be favored by the treatment of psoriasis with corticosteroids or novel immunosuppressive agents such as tumor necrosis factor alpha (TNFα) or interleukin-17 (IL-17) inhibitors, as these pharmacological interventions specifically affect antifungal immune responses [11]. Interestingly, psoriasin, which is produced in psoriatic plaques, has been shown to have broad antifungal activity by inducing apoptosis in several filamentous fungi, but it is not active against *C. albicans* [12].

## 2. *Candida* Pathogenesis at Epithelial Surfaces

Most of what is known about *C. albicans* pathogenesis on epithelial surfaces has been shown for the oral and vaginal mucosa, while less is known about skin invasion. The distinction between commensal colonization and invasive growth of *C. albicans* into epithelial surfaces is not clear cut, as adhesion to the host epithelium or even initial invasion may be relevant for establishment or maintenance of both states, while host cell damage is a distinct feature of pathogenic invasion [13,14]. Adhesion to epithelial cells has been studied for many *Candida* spp. and on different epithelial cell types, including fibroblasts, Caco-2 cells, buccal epithelial cells, and many others. Interestingly, cell surface adhesins in *Candida* spp. that colonize human epithelia like *Candida glabrata* or *Candida albicans* have developed in large gene families [15,16], indicating a specialization for colonization of the host. Extensive studies on binding motifs of lectin-like epithelial adhesins revealed that the different binding domains have well defined specificity for different di- or oligomeric carbohydrate combinations, enabling the colonization of a wide variety of host tissue [17,18]. For *C. albicans*, attachment to epidermal keratinocytes was also shown by direct interaction between moonlighting proteins [19] and host proteins, like the fungal protein Gpm1 (Phosphoglycerate mutase) and host cell vitronectin [20]. Two general phenomena can contribute to invasion of host epithelia: induced endocytosis and active penetration. *C. albicans* has been shown to induce its own passive uptake into normally non-phagocytic cells by a clathrin-dependent mechanism [21] that is dependent on Als3 or Ssa1 interaction with E-cadherin or N-cadherin expressed on epithelial host cells [22,23] or alternative pathways [24]. The contribution of induced endocytosis to epithelial invasion is, however, tissue or cell-type dependent. While induced endocytosis may potentially contribute to initial invasion of stratified epithelia such as the oral mucosa, intestinal enterocytes and Caco-2 cells do not internalize *C. albicans* by using this process. Here the fungus needs to actively penetrate the tissue [25,26]. In either case, active penetration is the predominant process of epithelial invasion [24]. In this case, the force is generated through a combination of hyphal extension and directional growth of the fungus [25]. Since the epidermis of skin is a cornified stratified epithelium shielded by a dense layer of dead keratinocytes, this barrier can likely only be breached by active penetration. Consistent with this notion, a non-hyphal *C. albicans* strain deleted for Efg1 and Cph1, the two major transcription factors involved in hyphae formation, is not able to invade intestinal or epidermal reconstituted epithelia [27]. In addition, this mutant cannot adhere to tissue due to a massive change in cell wall protein composition, and lacks expression of some key adhesins like ALS3 or HWP1 [26]. Besides being required for both active penetration and efficient induced endocytosis [25], hyphae formation is also a prerequisite for host tissue damage, a hallmark of *C. albicans* pathogenesis in epithelial tissues [8]. Physical force generated through hyphal extension as well as molecular factors such as secreted aspartyl proteases (SAPs) have been proposed to cause damage in an oral epithelial model, in particular Sap1-3 [28], while Sap1-2 have been associated with damage during invasion of a reconstituted vaginal epithelium [29]. Several Saps have also been shown to be overexpressed during *C. albicans* invasion of an epidermal model [30]. However, the precise contribution of Saps to *C. albicans* pathogenicity has been controversial. Additional results indicate that Saps are not required for invasion into reconstituted human oral or vaginal epithelia and that Sap1–6 are dispensable for virulence in a mouse model of disseminated candidiasis [31,32,33]. More recently, Candidalysin, a pore forming toxin, generated from the ECE1 gene product, which is expressed specifically during filamentous growth of *C. albicans*, was shown to contribute to damage of the oral epithelium during *C. albicans* invasion. Most interestingly, peptide 3, a short peptide derived from the Ece1p precursor molecule, has been shown to induce cell death by perforating the host cell membranes [34].

## 3. Initial Fungal Recognition

**Figure 1:**
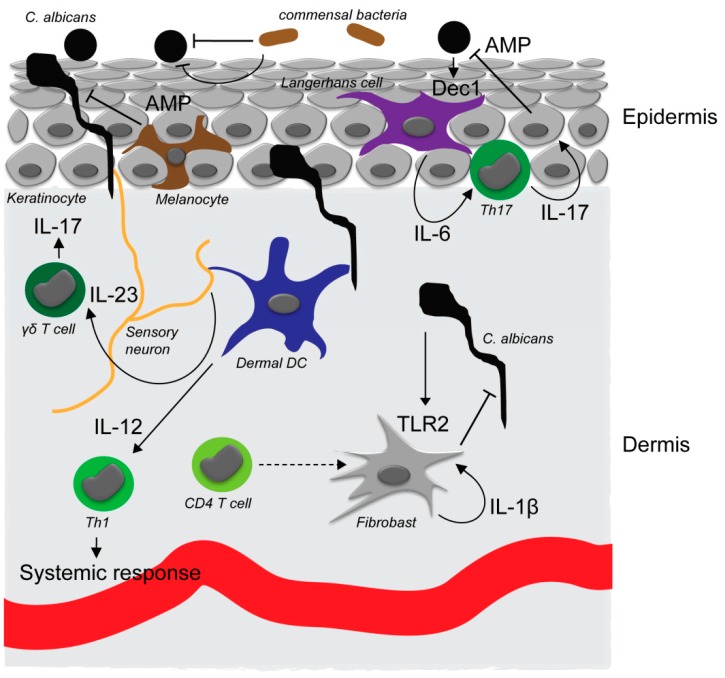
*C. albicans* interaction with the skin. In the commensal, noninvasive state, *C. albicans* exists in the yeast form on the tissue surface. Yeast cells can be detected through the dectin-1 receptor (Dec1) that is expressed by Langerhans cells in the epidermis. Activation of Langerhans cells (LCs) results in an interleukin-6 (IL-6)-dependent Th17 response, antimicrobial peptide production by keratinocytes and superficial antifungal defense. Commensal skin bacteria are also involved in preventing invasion of *C. albicans* into the skin by direct and indirect mechanisms. *C. albicans* invading the epidermis can moreover be detected by sensory neurons that promote IL-23 secretion by dermal dendritic cells and subsequent proliferation and IL-17 secretion of skin resident γδ T cells. Upon breaching of the epidermis, *C. albicans* is detected in the dermis by dermal dendritic cells which induce an IL-12-dependent Th1 response required for systemic immunity against the fungus. Dermal fibroblasts support direct antimicrobial defense in the dermis upon activation through TLR2 by *C. albicans* and IL-1β secretion and auto-activation. Secretion of IL-1β by dermal fibroblasts requires an additional yet unidentified signal from CD4^+^ T cells.

*Candida* invasion of the skin is rapidly recognized by innate immune receptors, such as pattern recognition receptors (PRRs), which initiate an efficient immune response (Figure 1). Different fungal structures such as the cell wall components β-glucans, mannans, and phospholipomannans can be sensed by receptors belonging to various classes of PRRs including Toll-like receptors (TLRs) and C-type lectins [35]. Their contribution to host defense against *Candida* species is, however, tissue specific and dependent on fungal morphology. Dectin-1 is a C-type lectin expressed on various cell types including monocytes, macrophages, dendritic cells, and neutrophils [36]. It recognizes the fungal cell wall component β-glucan and is involved in epithelial antifungal defense by Th17 induction in a Syk- and Card9-dependant manner [37]. β-glucan has been reported to primarily be surface accessible at the bud scars in the yeast form of *C. albicans* [38]. Hyphae are therefore thought to be invisible to dectin-1. *C. albicans* moreover reduces recognition by shielding the bud scars with a mannoprotein layer, thereby further limiting accessibility of β-glucan [38]. On the other hand, *C. albicans*-induced degranulation and TNF-α, IL-6, IL-10, CCL3, and CCL4 production by mast cells, which are also present in the skin, has been shown to be dectin-1-dependent, irrespective of the morphological state of *C. albicans*, while IL-1β production by mast cells was observed only in response to yeast cells [39]. Langerin, which is specifically expressed on epidermal Langerhans cells, was additionally proposed to play a central role in initial recognition of a broad spectrum of fungi including *C. albicans* on the skin. The authors could show that this C-type lectin binds not only to β-glucan, but also to mannose structures of fungal origin [40]. Activation of the inflammasome seems hyphae specific and likely involves recognition of mannan. *C. albicans* hyphae induce, for example, NLRP3-dependent inflammasome activation and IL-1β secretion by macrophages in the skin [8,41]. MDA5, an intracellular receptor for double-stranded RNA, has also recently been associated with *C. albicans* hyphal recognition by macrophages, and its induction upon fungal stimulation was reduced in peripheral blood mononuclear cells (PBMCs) from CMC patients [42]. It remains to be determined if there is a direct role of MDA5 in skin defense against *C. albicans* and what may be the fungal ligand for this receptor.

The precise role of individual TLRs in skin defense against *C. albicans* is not yet completely understood. The fungal cell wall components O-linked mannans and phospholipomannans (PLM) are detected by TLR4 and TLR2, respectively, and result in the activation of pro-inflammatory processes by signaling through MyD88 and NF-κB [43,44]. TLR2 and TLR4 are expressed on the surface of myeloid and lymphoid cells but also on epithelial cells. On keratinocytes, TLR2 expression has been shown repeatedly and TLR2-dependent activation of NF-κB and p38MapK in response to *C. albicans* PLM has been reported [45,46]. Moreover, in mouse epidermal Langerhans cells, MyD88 is necessary to respond to *C. albicans* invasion [47]. We have recently shown that among TLRs, TLR2 is most highly expressed in dermal fibroblasts in a CD4^+^ T cell supplemented human 3D skin model. We could further show that TLR2 is involved in dermal protection of this tissue model against *C. albicans* invasion in an IL-1β-dependent manner [48,49]. Moreover, TLR3, which recognizes nucleic acids, has also been associated with protection against cutaneous candidiasis [50,51]. TLR2 and TLR4 signaling is also involved in mucosal defense. TLR4 is induced in oral epithelial cells upon *C. albicans* infection of a reconstituted human epithelium in the presence of polymorphonuclear leukocytes and results in a direct epithelial antifungal defense [52]. In contrast, polymorphisms in TLR2, but not in TLR4 were associated with recurrent vulvovaginal candidiasis in humans [53].

## 4. Immune Networks Involved in Skin Defense against *Candida* Species

The subsequent defense reaction to a *Candia* infection also varies between different tissues [5]. In the skin, IL-17 is a central cytokine shaping immunity against *C. albicans* invasion of the epidermis. Mutations in five genes related to IL-17 and IL-17 signaling have been found to result in CMC in humans. These include deficiencies in IL-17 receptors: IL-17RA and IL-17RC, which are expressed on various cell types including epithelial cells [54] as well as IL-17F [55] which, like the prototypic IL-17A, binds to the heterodimeric IL-17RA/IL17-RC receptor [56]. In addition, deficiency of the adapter molecule ACT1 [57], and gain of function mutations in STAT1, lead to increased Th1 and Th2 responses and to a decrease in Th17 cells [58]. IL-17C is another IL-17 family member that can be detected in the skin. It is expressed by keratinocytes and together with IL-17A and IL-17F is involved in the pathology of psoriasis [59]. However, in contrast to IL-17RC, IL-17C and its receptor IL-17RE is not required for skin, oral, and systemic defense against *C. albicans* infection, as shown using *Il17c^−/−^*, *Il17re^−/−^*, and *Il17rc^−/−^* mouse models [59,60]. IL-17C or IL-17RE may therefore be interesting targets for the treatment of psoriasis without increasing susceptibility to CMC, as has been reported for IL-17A directed therapies, albeit at low incidence [61]. Th17 cells, which express IL-17A and IL-17F, play a crucial role in human defense against CMC. They are induced by *C. albicans* and are regulated by the proinflammatory cytokine IL-1β [62]. Antigen-presenting Langerhans cells (LCs) which reside in the epidermis have been shown to be sufficient to promote CD4^+^ T cell differentiation to Th17 cells in mice [63]. Upon skin infection with *C. albicans*, LCs are activated through TLR/MyD88 and dectin-1 signaling and secrete IL-6 which promotes Th17 differentiation [5,47]. While LCs are necessary for the development of antigen-specific Th17 cells, they are not crucial in the clearance of primary infections in an epicutaneous mouse model of candidiasis [64]. IL-17A and IL-17F are not solely produced by recruited Th17 cells, but also by skin resident γδ T cells which can sense invading pathogens through TLR2 and dectin-1 and allow for a fast defense reaction [54,65]. This has been shown to depend on IL-1β and IL-23, which are secreted by dendritic cells (DC) and lead to the expansion and secretion of IL-17A and IL-17F by γδ T cells [66,67]. IL-23-dependent IL-17A production has been shown to be critical for defense against *C. albicans* skin infection in an epicutaneous infection model using *Il17af^−/−^* and *Il23a^−/−^* mice [68], and humans with deficiencies in IL-23R signaling show increased susceptibility to CMC [69]. Interestingly, IL-23 secretion by CD301b^+^ dermal dendritic cells was found to depend on the neuropeptide CGRPα, which is secreted by nociceptive neurons directly sensing *C. albicans* in the skin [68]. This finding links the neuronal system to immunity against fungal skin infections.

While skin defense against *C. albicans* in the epidermis is predominantly governed by IL-17A/IL-17F-producing cells, protection of deeper tissue such as the dermis and prevention of systemic fungal dissemination has been reported to rely on a Th1 response [64]. Secretion of IL-6 by Langerhans cells and subsequent Th17 polarization depends on fungal recognition by dectin-1, which has been reported to be restricted to detect the yeast form of *C. albicans* only [64]. In contrast, neither LCs nor CD11b^+^ dermal DCs respond to *C. albicans* in its filamentous form, which is present in the dermis in a mouse skin infection model. Instead, the pseudohyphae are sensed by CD102^+^ dermal dendritic cells, leading to an induction of Th1 cells and protection against systemic *C. albicans* dissemination [64]. Using an immune cell supplemented human 3D skin model [48], we could show that activated CD4^+^ T cells contribute to dermal protection by inducing an antimicrobial response of dermal fibroblasts in the presence of *C. albicans*. Notably, this response in the dermal skin compartment was independent of IL-17A and IL-17RA signaling but relied on the induction of IL-1β secretion by dermal fibroblasts [49]. Besides Th17 and Th1 responses, skin resident and recruited Th9 cells have been reported to play a role in early defense against *C. albicans* skin infection, as reviewed in [70].

## 5. Antifungal Defense Execution by Antimicrobial Agents

Skin resident immune and non-immune cells as well as recruited immune cells are involved in the clearance of *C. albicans* in the skin. The first line of antifungal defense is carried out by keratinocytes, which produce antimicrobial peptides (AMPs) such as Cathelicidine/LL-37 and β-defensins and S100 proteins. LL-37 and human β-defensins 1 to 3 alter membrane integrity of *C. albicans* and provoke ATP release, but, in addition, alternative mechanisms of antifungal action have been described, as reviewed by [71]. Calprotectin, a dimer of S100A8 and S100A9, is able to inhibit *C. albicans* growth [72]. This effect is reversible by the addition of 30 µM zinc, indicating that the antifungal activity of calprotectin is likely through zinc chelation [73]. RNase7, which is released by keratinocytes, has been shown to exert candidacidal activity at low micromolar concentrations by membrane destabilization and targeting of fungal RNA upon internalization [74]. Antimicrobial peptides can be constitutively expressed in keratinocytes or regulated directly in response to PRR signaling or through inflammatory cytokines [75]. For example, IL-17F and mainly IL-17A induce the production of human β-defensin 2 and S100 proteins by primary human keratinocytes, and IL-22, which is also produced by Th17 cells, synergizes with these IL-17 cytokines in AMP induction by keratinocytes [76]. Moreover, together with TNF-α, IL-22 induces S100A7, human β-defensin 2, and the antimicrobial chemokines CXCL9, 10, and 11 in keratinocytes in vitro [77,78], yet the role of IL-22 in the defense of CMC is controversial [79]. CXCL9, 10, and 11 are also highly expressed in dermal fibroblasts during *C. albicans* infection of a CD4^+^ T cell supplemented human skin model [49], and CXCL10 has been shown to directly inhibit *C. albicans* growth in the radial diffusion assay [80]. 

IL-17 also contributes to *Candida* clearance at epithelial surfaces through recruitment of neutrophils, which kill fungal cells by releasing high amounts of antimicrobial peptides, by direct phagocytosis, or by formation of neutrophil extracellular traps (NETs) [5,81,82], but the role of neutrophils in epithelial antifungal defense has been reported to be tissue specific and therefore may not be generalized [83].

## 6. Fungal-Microbiome Interaction

It is becoming increasingly clear that local commensal microbial communities highly influence the mode of interaction of *C. albicans*—whether commensal or pathogenic—with epithelial surfaces. Treatment of mice with several classes of antibiotics has been shown to increase colonization of the intestine with *Candida* species and the intestinal bacterium *Bacteroides thetaiotamicron* has been observed to limit *C. albicans* colonization by increasing HIF-1α-dependent LL-37 expression [84]. Other bacteria, especially *Lactobacilli*, influence *C. albicans* colonization and invasion in the gastrointestinal tract and vaginal mucosa by various mechanisms including pH changes and modulation of the immune response [8]. Modulation of *Candida* infection of the skin is likely also highly influenced by skin commensals. For example, differentiated primary human keratinocytes were shown to sense secreted factors of the commensal bacterium *Staphylococcus epidermidis* through TLR2, leading to NF-κB activation. In combination with pathogenic *Staphylococcus aureus*, this results in the synergistic induction of the antimicrobial peptides β-defensin 3 and RNase7 [85]. Since both AMPs also have antifungal activity, it is likely that these staphylococci in combination also influence *C. albicans* skin infection. Using specific pathogen free mice, it was recently shown that colonization with *S. epidermidis* is also sensed by dermal dendritic cells. These resident immune cells then initiate the development of a CD8^+^ and IL-17^+^ T cell population. These cells are subsequently recruited to the epidermis, where they boost S100A8 and S100A9 production by keratinocytes, resulting in increased resistance against *C. albicans* invasion in the absence of inflammation [86].

## 7. Conclusions

The skin is similar to mucosal surfaces such as the oral and vaginal mucosa, as they are all stratified squamous epithelia. Yet there are also substantial differences, like different degrees of cornification in these tissues, and, importantly, their local environment. The mechanisms underlying skin colonization and invasion by *Candida* species as well as host defense mechanisms are consequently overlapping with those found for mucosal surfaces, but considerable differences such as the precise mode of pathogenic invasion, the role of different immune cell types in defense against *C. albicans*, and the influence of commensal microorganisms on *Candida* pathogenesis exist. Much has been learned from the analysis of genetic disorders in humans resulting in chronic mucocutaneous candidiasis and also from mouse models. These findings have revealed both mechanisms of fungal recognition as well as immunological networks required to control these infections. Simple, often unicellular in vitro models furthermore have revealed initial mechanisms of pathogenesis in *Candida* infections. Recently, complex 3D tissue models of human origin containing defined elements of the immune system have been shown to be particularly amenable for identifying and modeling aspects of antifungal defense of the human skin. These recent advances in setting up complex 3D-tissue models have shown great promise already and therefore will most likely offer additional approaches to dissect and study *Candida*-skin interactions in great detail in the near future.
